# Variation in ‘fast-track’ referrals for suspected cancer by patient characteristic and cancer diagnosis: evidence from 670 000 patients with cancers of 35 different sites

**DOI:** 10.1038/bjc.2017.381

**Published:** 2017-11-28

**Authors:** Y Zhou, S C Mendonca, G A Abel, W Hamilton, F M Walter, S Johnson, J Shelton, L Elliss-Brookes, S McPhail, G Lyratzopoulos

**Affiliations:** 1 Cambridge Centre for Health Services Research, Primary Care Unit, Department of Public Health and Primary Care, University of Cambridge, Strangeways Research Laboratory, 2 Wort’s Causeway, Cambridge CB1 8RN, UK; 2 University of Exeter Medical School (Primary Care), Smeall Building, St Luke’s Campus, Exeter EX1 2LU, UK; 3 National Cancer Registration and Analysis Service, Public Health England Zone A, 2nd Floor, Skipton House, 80 London Road, London SE1 6LH, UK; 4 Cancer Research UK, Angel Building 407 St John Street, London EC1V 4AD, UK; 5 Epidemiology of Cancer Healthcare and Outcomes (ECHO) Research Group, Department of Behavioural Science and Health, University College London, 1-19 Torrington Place, London WC1E 7HB, UK

**Keywords:** cancer diagnosis, diagnostic pathway, cancer epidemiology, primary care, early diagnosis

## Abstract

**Background:**

In England, ‘fast-track’ (also known as ‘two-week wait’) general practitioner referrals for suspected cancer in symptomatic patients are used to shorten diagnostic intervals and are supported by clinical guidelines. However, the use of the fast-track pathway may vary for different patient groups.

**Methods:**

We examined data from 669 220 patients with 35 cancers diagnosed in 2006–2010 following either fast-track or ‘routine’ primary-to-secondary care referrals using ‘Routes to Diagnosis’ data. We estimated the proportion of fast-track referrals by sociodemographic characteristic and cancer site and used logistic regression to estimate respective crude and adjusted odds ratios. We additionally explored whether sociodemographic associations varied by cancer.

**Results:**

There were large variations in the odds of fast-track referral by cancer (*P*<0.001). Patients with testicular and breast cancer were most likely to have been diagnosed after a fast-track referral (adjusted odds ratios 2.73 and 2.35, respectively, using rectal cancer as reference); whereas patients with brain cancer and leukaemias least likely (adjusted odds ratios 0.05 and 0.09, respectively, for brain cancer and acute myeloid leukaemia). There were sex, age and deprivation differences in the odds of fast-track referral (*P*<0.013) that varied in their size and direction for patients with different cancers (*P*<0.001). For example, fast-track referrals were least likely in younger women with endometrial cancer and in older men with testicular cancer.

**Conclusions:**

Fast-track referrals are less likely for cancers characterised by nonspecific presenting symptoms and patients belonging to low cancer incidence demographic groups. Interventions beyond clinical guidelines for ‘alarm’ symptoms are needed to improve diagnostic timeliness.

Most patients with cancer first present with symptoms, typically to a general practitioner (GP) in primary care (Elliss-Brookes *et al*, 2012). Reducing diagnostic delay in these patients by addressing contributory patient and healthcare system factors is an important goal for healthcare systems (Walter *et al*, 2012). In England, dedicated referral pathways supported by national clinical guidelines have been in existence since 1999 to expedite referral for the diagnostic evaluation of patients with suspected cancer in order to reduce the length of diagnostic intervals after patients present to their GPs (NICE, 2005). Patients judged to meet criteria for such referrals are offered ‘urgent’ assessment by specialist hospital services within 2 weeks. Hereafter, we use the term ‘fast-track’ to denote referrals through this pathway, otherwise known as the ‘two-week-wait’ or ‘urgent’ referral pathway for suspected cancer. Although diagnostic delays may occur both because of patient and system factors (i.e., before and after presentation), in this paper we focus on variation in the type of GP referral route, as a factor that can affect the length of the post-presentation intervals.

Fast-track referral criteria are typically based on the presence of ‘alarm’ symptoms, although many cancer patients have no such presenting symptoms (Stapley *et al*, 2012; Howell *et al*, 2013; Din *et al*, 2015). Consequently, large proportions of patients with cancer continue to be diagnosed through other diagnostic routes, including following an emergency presentation, which is associated with poorer survival (Elliss-Brookes *et al*, 2012; Abel *et al*, 2015). Among cancer patients who are diagnosed after a GP referral, nearly half are diagnosed after non-fast-track referrals (Elliss-Brookes *et al*, 2012; NCIN, 2015). Despite a continual increase in fast-track referral activity from 0.9 million to over 1.5 million between 2009–2010 and 2014–2015 (NCIN, 2016), and evidence suggestive of associations between higher general practice fast-track referral activity and cancer survival (Møller *et al*, 2015), fewer than half of all cancer patients are diagnosed through this route. Therefore, considering how this proportion can be further increased is important.

Previous research has examined practice-level variation in fast-track referrals (Rogers *et al*, 2014; Maclean *et al*, 2015; Murchie *et al*, 2015), but there is limited understanding of variation in the type of primary–secondary care referrals between different patient groups once a GP has decided to refer. Among patients who have seen their doctor, understanding differences in the frequency of fast-track and non-fast-track referrals can provide insights into factors that influence decision making by GPs about the referral of symptomatic patients, and enable the design and implementation of targeted interventions to further increase the proportion of cancer patients diagnosed who were fast-tracked. Such analyses can also provide a ‘real-life’ evaluation of the effectiveness of healthcare policies supporting fast-track referral pathways for suspected cancer and identify the need for alternative strategies. We therefore examined variation in referral type among patients whose diagnosis involved either a fast-track or an elective GP referral, to examine factors that might influence a GP’s decision to refer through either of these routes.

## Materials and methods

### Data

Data were extracted from the National Cancer Data Repository (NCDR) containing information on the diagnostic ‘route’ of all patients diagnosed with cancer in England during the study period (2006–2010). ‘Diagnostic routes’ represent care pathways to diagnosis. They are assigned using an algorithm based on linked data from cancer registration, Hospital Episodes Statistics, National Cancer Waiting Times and National Health Service Cancer Screening Programmes (for breast, bowel and cervical cancers) as previously described (Elliss-Brookes *et al*, 2012).

We only included patients who were diagnosed through a ‘two-week wait’ and ‘non-two-week wait’ GP referral, excluding patients diagnosed through any other diagnostic route (i.e., after screening, elective hospitalisation, hospital outpatient appointment (other than after a GP referral), emergency presentation, unknown route or following death certification only). We did so because we were *a priori* interested in factors that make the suspicion of cancer diagnosis either harder or easier once patients have consulted with a GP, outside of circumstances where an emergency hospital referral is needed.

The ‘two-week wait’ route denotes diagnosis after an urgent GP referral with a suspicion of cancer through the fast-track pathway, whereas the non-two-week wait route represents elective GP referrals that do not fit the fast-track criteria.

Available data included information on all patients with one of 35 categories of malignant neoplasms including anal, bladder, breast, breast carcinoma *in situ*, colon, oral, oropharyngeal, laryngeal, Hodgkin lymphoma, four types of leukaemia (acute lymphoblastic leukaemia (ALL), acute myeloid leukaemia (AML), chronic lymphocytic leukaemia (CLL) and chronic myeloid leukaemia (CML)), liver, lung, malignant brain, melanoma, mesothelioma, multiple myeloma, non-Hodgkin lymphoma, oesophageal, ovarian, pancreatic, penile, prostate, rectal, renal, soft tissue sarcoma, small intestine, stomach, testicular, thyroid, uterine, vulval and cancer of unknown primary. Data were also available on the patient’s age group, sex, year of diagnosis and deprivation group based on national quintiles of the income domain of the Index of Multiple Deprivation 2010 of the lower super output area of the patients’ postcode of residence (Department of Communities and Local Government, 2011).

### Analysis

#### Main effect analysis

We examined the differences in crude proportions of patients diagnosed through the fast-track route, by cancer site, age, sex, deprivation group and year of diagnosis. The choice of these variables reflects our hypothesis that among patients who were diagnosed with cancer following a GP referral, the use of fast-track referral varies by patient characteristic and cancer site. Subsequently, we used logistic regression to estimate odds ratios for fast-track route by variable category, first unadjusted, then adjusted for age, sex, deprivation, year of diagnosis and cancer site (for the latter, rectal cancer was used as the reference category as a common cancer in either sex). Because the adjusted odds ratios derived by the multivariate model do not directly translate to the natural scale and to aid interpretation of the findings, we additionally used the model output to estimate the adjusted proportions of fast-tracked patients for each variable group using the marginal standardisation method (Muller and MacLehose, 2014).

#### Effect modification analysis

Because of prior evidence documenting effect modification between patient characteristics and cancer diagnosis in respect of other markers of diagnostic difficulty (e.g., the number of pre-referral consultations and the proportion of emergency presentations), we additionally examined interactions between each of the three sociodemographic variables (age, sex and deprivation) and cancer in respect of odds of fast-track referrals (Lyratzopoulos *et al*, 2012; Abel *et al*, 2015). We added all three interaction terms to the main effects model and retained those that tested significant (*P*<0.001) using the joint Wald test.

## Results

There were 669 220 incident tumours contained within the 35 cancer sites diagnosed through either category of GP referrals between 2006 and 2010. Of these 339 500 (51%) were diagnosed after fast-track and 329 720 (49%) after non-fast-track referrals.

Hereafter, the patterns of variation described relate to cancer patients diagnosed following a GP referral (either fast-track or non-fast-track) only, and do not relate to ‘every’ patient in the population with those cancers, excluding, for example, patients diagnosed through screening, or after an emergency presentation (see Data in Materials and Methods).

### Main effects analysis

**Figure 1:**
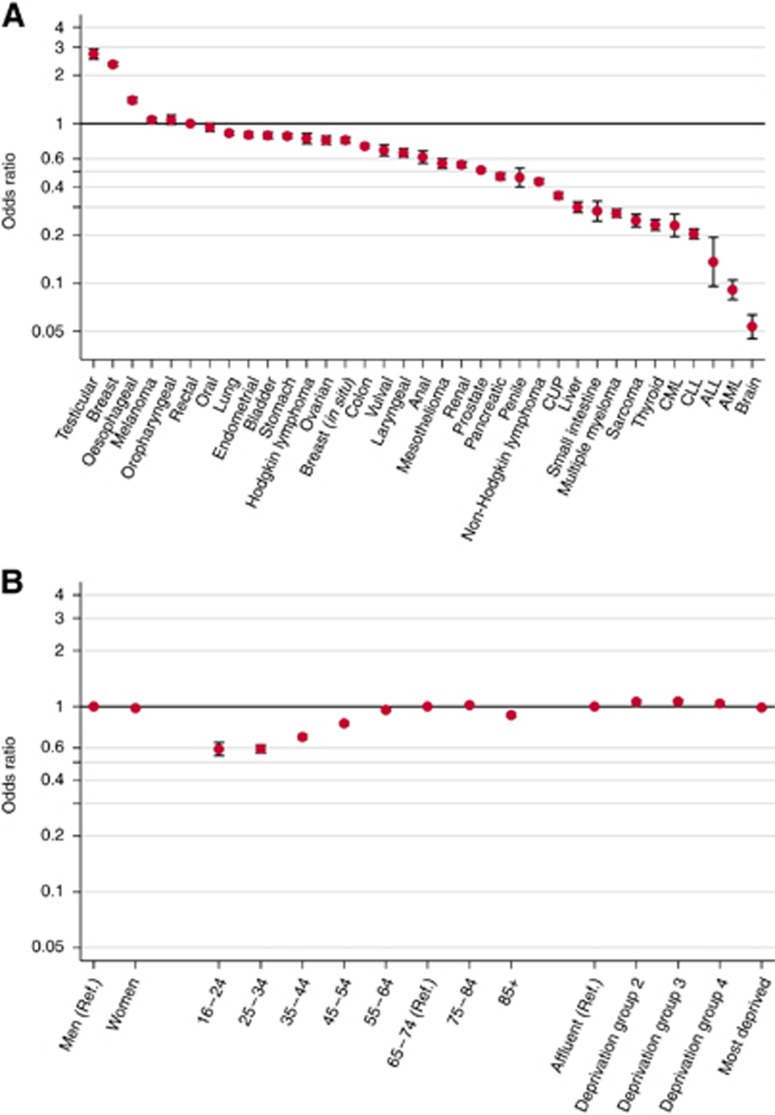
**Main effect variations in adjusted odds ratios of fast-track referral.** (**A**) Variation by cancer. (**B**) Variation by sociodemographic characteristic. ALL=acute lymphoblastic leukaemia; AML=acute myeloid leukaemia; CLL=chronic lymphocytic leukaemia; CML=chronic myeloid leukaemia.

Considering unadjusted analyses, there was very strong evidence (*P*<0.0001) for very large variation in the proportions of fast-track referral by cancer site. This proportion was highest (73 and 71%) for patients with breast and testicular cancer, respectively, and lowest (6%) for patients with brain cancer. In multivariable analysis, the results were largely concordant with those of the unadjusted analysis. Patients subsequently diagnosed with testicular and breast cancers were most likely to be diagnosed through the fast-track pathway (OR 2.73, 95% CI 2.54–2.93 testicular *vs* rectal cancer, Table 1 and Figure 1A). Patients subsequently diagnosed with brain cancer and any type of leukaemia (AML, ALL, CLL, CML) were the least likely to be referred through the fast-track route (OR 0.05, 95% CI 0.05–0.06, *P*<0.0001; brain *vs* rectal cancer). The respective adjusted proportions for patients with testicular and brain cancer were 77% and 6%. To appreciate the very large size of variation by cancer site it should be noted that there is >50-fold variation in the adjusted odds of fast-track referrals between testicular and brain cancer, with a respective 12-fold variation in adjusted proportions.

There was also very strong evidence (*P*<0.0001 for age, sex and deprivation) of variation in the crude proportions of cancer patients who were diagnosed after fast-track referral for patient characteristics, although the size of sociodemographic variation, where present, was relatively small compared with that observed for cancer site. Specifically, there were moderate differences in the proportion of fast-track referrals by age group and sex (Table 1), for example, 47 *vs* 50% for 25–34/65–74 year olds and 46 *vs* 56% for men/women. There was little variation in fast-track referral proportions by deprivation group, with slightly lower proportions of fast-track referrals in the highest and lowest quintiles. The proportion of referred patients who were fast-tracked increased during the 5-year study period from 48% in 2006 to 53% in 2010 (*P*<0.0001). In multivariable analysis, there was strong evidence for variation by age group, with increasing odds of fast-track referral up to age 84 years (*P*<0.0001, Table 1 and Figure 1B). Although women had a higher chance of being fast-tracked than men in the unadjusted analysis, a much smaller, and inverse, association was found in adjusted analysis (OR 0.98, 95% CI 0.97–1.00, *P*=0.013). This observation reflects that breast cancer, that has the highest proportion of fast-track referrals compared with all cancers sites (73%), is far more common among women; once cancer site is controlled for, this crude sex difference is adjusted accordingly. The variation by deprivation group remained limited in the unadjusted and adjusted analyses. The odds of fast-track referrals were greater in 2010 compared with 2006 (adjusted OR 1.28, 95% CI 1.26–1.30, *P*<0.0001).

### Effect modification

**Figure 2:**
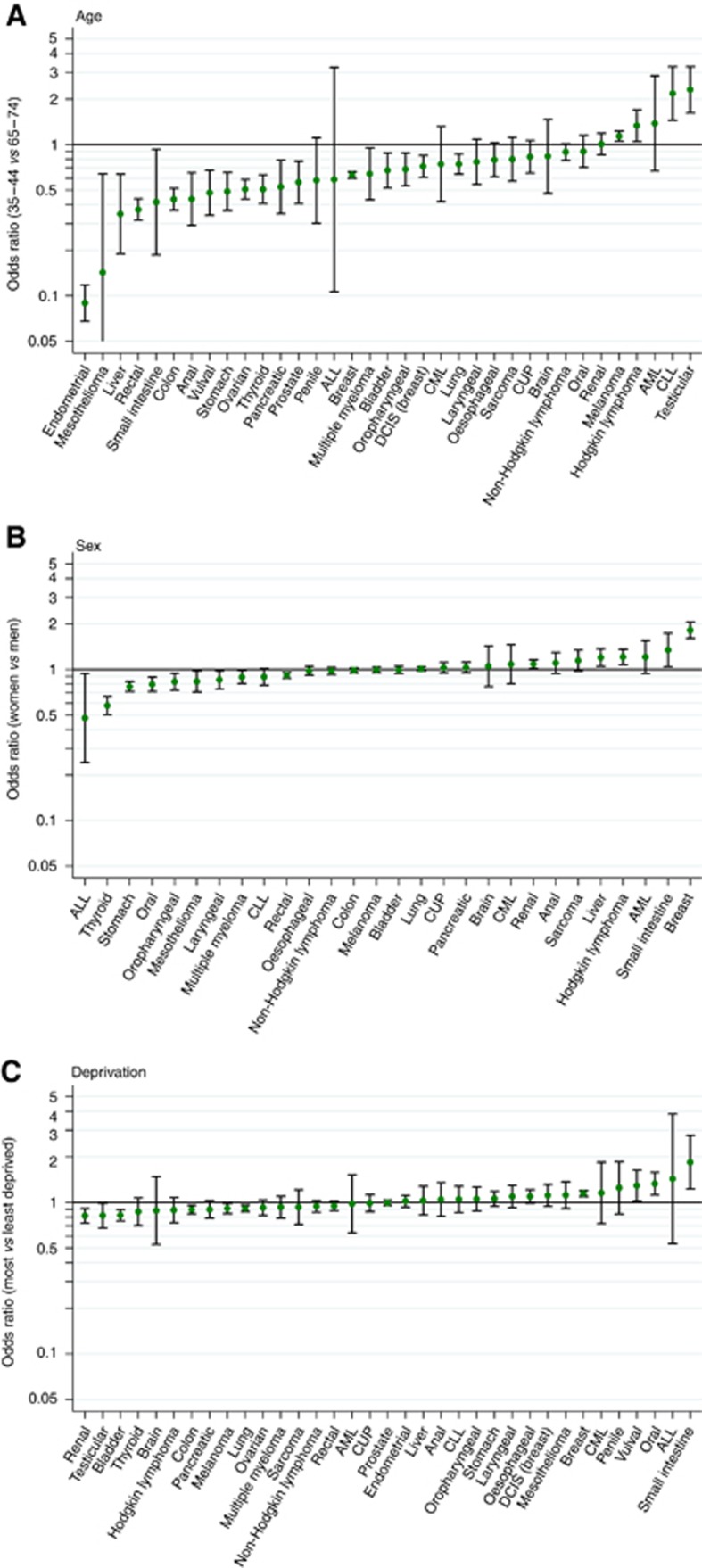
**Cancer-specific odds ratios and associated 95% confidence intervals for fast-track referrals, comparing:** (**A**) 35–44 year olds with 65–74 year olds; (**B**) Women with men; (**C**) Most with least deprived. ALL=acute lymphoblastic leukaemia; AML=acute myeloid leukaemia; CLL=chronic lymphocytic leukaemia; CML=chronic myeloid leukaemia.

There was strong evidence for interactions between cancer diagnosis and age, cancer diagnosis and sex, and cancer diagnosis and deprivation (Figure 2A–C; *P*<0.0001 for all), meaning that associations between patient characteristics and odds of fast-track referral varied notably for patients with different cancers (specific examples discussed in Box 1). The cancer-specific variation was largest for age and much smaller for sex and deprivation (Figure 2A–C).

## Discussion

We document large variations by cancer diagnosis and patient characteristics in the proportion of patients who were diagnosed after a fast-track primary-to-secondary care referral. Associations of age, sex and deprivation varied in size and direction for patients with different cancers.

### Comparison with previous studies

Our findings amplify previous reports describing crude proportions of fast-track referral pathways for patients with 15 and 38 cancers (Elliss-Brookes *et al*, 2012; NCIN, 2014), but the use of multivariable analysis allowed us to estimate independent associations between fast-track referral and cancer site, age, sex and deprivation group. These independent associations are not subject to confounding by any of the attributes included in the model. To illustrate the impact of confounding one can consider that the highest unadjusted proportion of fast-track referrals by age was observed in patients aged 35–54 years (Table 1), reflecting that ∼40% of cancer patients of that age in our analysis sample are women with breast cancer. However, after adjustment for cancer site (and other variables) this pattern of variation by age is lost. Furthermore, for the first time we report on effect modification of patient characteristics by cancer site in respect of the odds of fast-track referral. The increasing proportion of patients diagnosed after a fast-track referral during the study period suggests that the recently reported increase in ‘fast-track’ activity between 2010 and 2014 may have started during 2006–2010 (Elliss-Brookes *et al*, 2012; NCIN, 2016). This trend could be a reflection of the influence of healthcare interventions aimed at improving cancer diagnosis, such as the introduction of clinical guidelines for fast-track referrals for suspected cancer (NICE, 2005).

### Strengths and limitations

We used data from a large population-based sample that employed robust methodologies to assign diagnostic route and included a wide range of cancers (Elliss-Brookes *et al*, 2012). We did not have information on the general practice and presence of co-morbidities of the referred patients, and therefore in part our findings may be confounded by variation between practices and patients (e.g., if practices with predominantly older patients have higher ‘fast-track’ referral rates, then the age patterns that we report may be either overestimating or underestimating the true variation by age). However, the reported confidence intervals and *P-*values emanating from our regression models are robust to potential overdispersion.

Although appropriately used in literature examining variations in cancer processes and outcomes, odds ratios may lead to an exaggerated perception of the size of differences in odds of fast-track referrals between different patient groups. Although there is a 50-fold variation in the adjusted odds of a fast-track referral between cancer sites, the corresponding variation in adjusted proportions is 12-fold (see Table 1, ‘Adjusted proportions’ column). Nonetheless, this is still a very large difference by cancer site in the proportion of patients diagnosed through a fast-track referral.

We had no information on the diagnostic interval. However, it can be assumed that referral intervals (from date of referral to date seen at hospital) for most ‘routinely’ referred patient would be longer by a few weeks compared with most patients who were referred as ‘two-week wait’ referrals. Although our study only examined post-presentation referral pathways, improvements towards earlier diagnosis can also be achieved by shortening both pre-presentation (mainly relating to patient factors) and post-presentation intervals (patient and system factors) (Lyratzopoulos *et al*, 2014, 2015).

Lastly, although we focus on patients who were referred through the fast-track or elective route, approximately a fifth of all emergency presentations (∼3% of all incident cases) would occur while the patient is awaiting to be seen following a referral to hospital services (Murchie *et al*, 2017). Those patients form part of about two-third of emergency presenters who have had at least one prior relevant GP contact preceding their emergency presentation (Abel *et al*, 2017; Murchie *et al*, 2017; Zhou *et al*, 2017). Our study population relates to cancer patients who have been referred by their GP. As such, the observed associations should not be taken to relate to all patients who presented to a GP and were subsequently diagnosed with cancer. Our study provides insights into GP decision making regarding the use of different type of referrals once a decision to refer has been made. A proportion of cancer patients are diagnosed following a GP consultation who did not result in either an urgent or elective referral; and our findings do not extend to this group.

### Interpretation and implications

In considering the implications of the findings, we focus on patient groups at the extremes of the spectrum of fast-track referral variation where inferences can be more reliable, highlighting possible mechanisms for the observed variation, and describing possible clinical, policy or research implications. We consider two principal mechanisms that may underlie the observed variation.

#### 1. Symptom signature (variation by cancer in proportions of patients with ‘alarm’ symptoms, and vice versa)

Cancer patients most likely to be fast-tracked include those with testicular, breast, oesophageal, melanoma, oropharyngeal, oral and endometrial cancer, where ‘alarm’ symptoms (such as testicular or breast lumps, dysphagia, visible skin abnormalities, oral ulceration and vaginal bleeding, respectively) are present in most patients at presentation. Conversely, patients with cancers least likely to be diagnosed after fast-track referrals encompass brain cancer, the leukaemias and multiple myeloma, all characterised by presenting with symptoms of low predictive value in most patients. For example, approximately half of all patients with multiple myeloma present with musculoskeletal/back pain, a common symptom with low specificity for cancer in primary care (Hamilton and Kernick, 2007; Shephard *et al*, 2015). Similarly, most patients with brain cancer and leukaemia, the two cancers dominated by high rates of emergency presentations (Abel *et al*, 2015), either present as a clinical emergency (such as a seizure or sepsis) or have vague presenting symptoms that may limit the use of fast-track referral pathways (Howell *et al*, 2013). For leukaemia, the availability of primary care testing (e.g., full blood count) may often result in an emergency admission through on-call haematology services, negating the need (or even the opportunity) for fast-track referral. The findings therefore further substantiate previous evidence indicating that cancers with an obvious symptom signature (i.e., where most patients present with symptoms of high enough predictive value) are more likely to be fast-tracked, and vice versa (Lyratzopoulos *et al*, 2012, 2013).

#### 2. Variation in the positive predictive values of symptoms between patient subgroups with the same cancer

Between patients with a given cancer, referral decisions seem to be influenced by variation in the predictive value of potential cancer symptoms in patients of different age and sex. This can also be seen as a reflection of the variable incidence of different cancers in patients of different demographic groups. For example, women with endometrial cancer who are older than 55 years have a notably higher fast-track referral rate compared with women under 55 years of age with the same cancer. A likely explanation of this pattern is that vaginal bleeding has a high predictive value for endometrial cancer in post-menopausal women, but is not an alarm symptom for young women. Conversely, testicular lump is likely to have higher predictive value for testicular cancer in young as opposed to older men, and we see that older patients with testicular cancer are much less likely to be referred urgently (Figure 2A). Therefore, the cancer-specific variations observed by age and sex likely reflect the underlying risk of a person of a particular age and sex to have that cancer.

Another example of the influence of variable predictive values of given symptoms in patients of different sociodemographic groups is provided by the variation by sex (women being less likely to have been fast-track referred) for thyroid cancer: These patterns may at least partly reflect the greater incidence of benign thyroid conditions associated with a lump/palpable thyroid gland swelling, including hypo- and hyperthyroidism in women compared with men (Vanderpump, 2011)).

Age restrictions in the fast-track guidelines may explain some of the cancer-specific variations by age for few cancers. For example, we see an increase in fast-track rate for breast cancer and endometrial cancer around the respective age thresholds for referral (30 years for breast, and ‘post-menopausal’ for endometrial); however the influence of age-specific referral thresholds is much less apparent for other cancers (Supplementary File 2). It is worth noting the inherent relationship between age cutoffs used in referral guidelines and the age-related PPV values *per se*. Fully disentangling the effects of the two is challenging.

Additionally, we reflect on other patterns of variation that may have clinical or policy implications but do not conform to the above two principal aetiological explanations (Box 2).

Good ‘safety netting’ practices (CRUK, 2014; Nicholson *et al*, 2016), patient education and engagement in monitoring of nonspecific symptoms, and clinical decision tools to assess risk of multiple nonalarm symptoms may be useful in reducing avoidable delays in cancers with difficult symptom signatures or patients in low risk strata.

In conclusion, among patients who were diagnosed following a GP referral, the use of fast-track referral varies substantially between patients with different cancers and sociodemographic characteristics. In particular, cancers with nonspecific symptoms and low-risk patients are less likely to have a fast-track referral. We have highlighted two possible mechanisms that may explain these variations, that is, variation by cancer in the proportion of patients with typical ‘alarm’ symptoms supported by referral guidelines; and in demographic groups with lower cancer risk. Recently, the National Institute for Clinical Excellence (NICE) in the United Kingdom has lowered cancer risk thresholds at which fast-track referrals are recommended from 5 to 3%, although the effects of this initiative are yet to be known (NICE, 2015). Our findings provide the basis for further research to examine how GPs make decisions in the diagnostic process, and motivate the development, evaluation and implementation of strategies to improve the timeliness of cancer diagnosis. These may include the use of guidelines that enable referrals or investigations at lower risk (possibly supported by clinical decision tools), the wider availability of one-stop multi-specialty diagnostic clinics and safety netting and patient activation interventions for particular cancer groups.

## Ethics

This study used anonymised aggregated data from the Routes to Diagnosis data set, available from the National Cancer Registration and Analysis Service, Public Health England. Individual patient consent and ethics clearance were therefore not required for this study.

## Figures and Tables

**Table 1:** Observed and adjusted proportions of fast-track referrals, and crude and adjusted odds ratios for fast-track referrals by cancer diagnosis, age, sex, year of diagnosis and deprivation group

		Fast-track referrals				
Variable category	Number of patients diagnosed through GP referrals	*N*	Observed (%)	Case-mix adjusted (%)^a^	Crude odds ratios^b^	Adjusted odds ratios^a,b^
**Cancer type**						
All cancers	669 220	339 500	50.7			
Testicular	6096	4346	71.3	77.2	1.94 (1.81, 2.08)	2.73 (2.54, 2.93)
Breast	112 962	82 787	73.3	74.5	2.14 (2.08, 2.21)	2.35 (2.28, 2.42)
Oesophageal	18 324	11 793	64.4	63.6	1.41 (1.35, 1.47)	1.40 (1.34, 1.46)
Melanoma	37 105	20 516	55.3	56.9	0.97 (0.93, 1.00)	1.06 (1.02, 1.09)
Oropharygeal	5291	2975	56.2	56.9	1.00 (0.94, 1.08)	1.05 (0.99, 1.13)
Rectal	35 452	19 906	56.1	55.6	Reference	Reference
Oral	6078	3276	53.9	54.3	0.91 (0.85, 0.98)	0.95 (0.89, 1.01)
Lung	74 553	39 533	53.0	52.2	0.88 (0.85, 0.91)	0.87 (0.85, 0.90)
Endometrial	24 431	12 656	51.8	51.6	0.84 (0.81, 0.87)	0.85 (0.82, 0.88)
Bladder	25 820	13 481	52.2	51.2	0.85 (0.82, 0.89)	0.84 (0.81, 0.87)
Stomach	13 739	7163	52.1	51.3	0.85 (0.81, 0.89)	0.84 (0.80, 0.88)
Hodgkin lymphoma	4578	2030	44.3	50.2	0.62 (0.58, 0.67)	0.81 (0.75, 0.87)
Ovarian	13 918	6766	48.6	49.6	0.70 (0.66, 0.75)	0.79 (0.74, 0.83)
Breast *in situ*	7023	3330	47.4	49.6	0.74 (0.70, 0.77)	0.79 (0.75, 0.82)
Colon	46 012	22 264	48.4	47.6	0.73 (0.71, 0.76)	0.72 (0.70, 0.75)
Vulval	3506	1597	45.6	46.0	0.65 (0.60, 0.71)	0.68 (0.63, 0.74)
Laryngeal	6522	2975	45.6	45.1	0.65 (0.61, 0.69)	0.66 (0.62, 0.70)
Anal	2822	1223	43.3	43.6	0.60 (0.54, 0.66)	0.62 (0.56, 0.67)
Mesothelioma	4742	2023	42.7	41.4	0.58 (0.54, 0.63)	0.56 (0.52, 0.60)
Renal	15 248	6268	41.1	40.9	0.55 (0.52, 0.57)	0.55 (0.53, 0.58)
Prostate	116 164	47 037	40.5	39.1	0.53 (0.52, 0.55)	0.51 (0.50, 0.53)
Pancreatic	11 016	4160	37.8	37.0	0.47 (0.45, 0.50)	0.47 (0.44, 0.49)
Penile	1244	458	36.8	36.6	0.46 (0.40, 0.52)	0.46 (0.40, 0.53)
Non-Hodgkin lymphoma	25 192	8864	35.2	35.3	0.42 (0.41, 0.44)	0.43 (0.42, 0.45)
Cancer of unknown primary	12 355	3829	31.0	30.8	0.35 (0.33, 0.37)	0.35 (0.34, 0.37)
Liver	4838	1352	27.9	27.4	0.30 (0.28, 0.33)	0.30 (0.28, 0.32)
Small intestine	1312	348	26.5	26.3	0.28 (0.24, 0.33)	0.28 (0.25, 0.33)
Multiple myeloma	8567	2251	26.3	25.7	0.28 (0.26, 0.30)	0.27 (0.26, 0.29)
Sarcoma	3607	822	22.8	23.7	0.23 (0.21, 0.25)	0.25 (0.22, 0.27)
Thyroid	6355	1267	19.9	22.6	0.19 (0.18, 0.21)	0.23 (0.21, 0.25)
CML	1133	246	21.7	22.5	0.22 (0.18, 0.26)	0.23 (0.20, 0.27)
CLL	6877	1440	20.9	20.4	0.21 (0.19, 0.22)	0.20 (0.19, 0.22)
ALL	399	48	12.0	14.7	0.11 (0.07, 0.15)	0.14 (0.10, 0.19)
AML	2800	293	10.5	10.3	0.09 (0.08, 0.11)	0.09 (0.08, 0.10)
Brain	3139	185	5.9	6.3	0.05 (0.04, 0.06)	0.05 (0.05, 0.06)
**Age**						
0–24	4421	1666	37.7	40.2	0.62 (0.54, 0.70)	0.59 (0.54, 0.64)
25–34	11 420	5361	46.9	40.3	0.90 (0.83, 0.97)	0.59 (0.56, 0.62)
35–44	34 449	18 261	53.0	43.5	1.15 (1.10, 1.20)	0.68 (0.66, 0.71)
45–54	68 321	36 402	53.3	47.4	1.16 (1.12, 1.20)	0.81 (0.79, 0.83)
55–64	134 733	67 668	50.2	51.2	1.03 (1.00, 1.06)	0.96 (0.94, 0.98)
65–74	188 210	93 298	49.6	52.2	Reference	Reference
75–84	172 467	88 726	51.4	52.6	1.08 (1.05, 1.11)	1.02 (1.00, 1.04)
85+	55 199	28 118	50.9	49.7	1.06 (1.02, 1.10)	0.90 (0.88, 0.92)
**Sex**						
Men	346 840	159 026	45.8	50.9	Reference	Reference
Women	322 380	180 474	56.0	50.5	1.50 (1.47, 1.53)	0.98 (0.97, 1.00)^c^
**Year of diagnosis**						
2006	124 593	59 746	48.0	47.7	Reference	Reference
2007	128 045	64 983	50.8	50.6	1.12 (1.08, 1.15)	1.14 (1.12, 1.16)
2008	134 180	68 358	50.9	50.9	1.13 (1.09, 1.16)	1.15 (1.13, 1.17)
2009	139 961	70 667	50.5	50.8	1.11 (1.07, 1.14)	1.14 (1.12, 1.17)
2010	142 441	75 746	53.2	53.3	1.23 (1.20, 1.27)	1.28 (1.26, 1.30)
**Deprivation quintile**						
1 (Least deprived)	133 976	66 688	49.8	50.0	Reference	Reference
2	147 422	75 604	51.3	51.4	1.06 (1.03, 1.09)	1.06 (1.04, 1.08)
3	143 681	74 018	51.5	51.4	1.07 (1.04, 1.10)	1.07 (1.05, 1.08)
4	130 559	66 611	51.0	50.9	1.05 (1.02, 1.08)	1.04 (1.02, 1.06)
5 (Most deprived)	113 582	56 579	49.8	49.7	1.00 (0.97, 1.03)	0.99 (0.97, 1.01)

Abbreviations: ALL=acute lymphoblastic leukaemia; AML=acute myeloid leukaemia; CLL=chronic lymphocytic leukaemia; CML=chronic myeloid leukaemia; GP=general practitioner.

a Estimates derived from model including main effect terms for cancer site, age, sex, deprivation and year of diagnosis.

b *P*<0.0001 for all based on joint Wald test of categorical variables, except for sex.

c *P*=0.0129 for adjusted odds ratios for sex.

## Box 1: Specific examples of cancer-specific variations by age, sex and deprivation

*Cancer-specific variation by age*
Illustrating variability in the size of the age association, there was a much steeper than average positive age gradient for women with endometrial cancer, with the odds of fast-track referral for young women being particularly small compared with older women with this cancer (OR 0.09, 95% CI 0.07–0.12, *P*<0.0001; 35–44 *vs* 65–74 year olds). Among all women with endometrial cancer, those ⩽55 years old comprise 3318 out of 24 431 cases (14%) in our sample. At the other end of the spectrum, *decreasing* odds of fast-track referral with increasing age were observed for testicular cancer, with younger men having appreciably higher odds of fast-track referral compared with older patients with this cancer (OR 2.31, 95% CI 1.62–3.28, *P*<0.0001; 35–44 *vs* 65–74 year olds, Figure 2A) – that is, a particularly steep negative age gradient. It should be noted that among all men with testicular cancer in our sample, those aged ⩾65 years comprise 211 of 6096 cases (3%).
Given the size of the interaction between age and cancer, we further estimated the odds of fast-track referrals by age group separately for each of the 35 cancers (Supplementary File 1). By and large, four patterns of associations can be distinguished.

1. An overall positive monotonic association (increasing age/increasing odds): anal, breast, prostate and vulval cancers;
2. A slow-rising increase in odds until late middle age, subsequently decreasing in older ages (an ‘n’ (or ‘inverse U’) shape pattern): bladder, colon, lung, ovarian, rectal and stomach cancer (‘peak’ in patients aged 55–64 years) and colon and rectal cancer (‘peak; in the 65–74 years);
3. A ‘flat’ pattern where the odds of fast-track referral remain similar across all age groups, acknowledging that in some cancers this pattern may also partially reflect small number of cases and hence very large confidence intervals around the estimate: for example, oral, oropharyngeal cancer, sarcoma and non-Hodgkin lymphoma.
4. An inverse (negative) association of decreasing odds with increasing age is observed: for example, testicular (see above), CLL.

*Cancer-specific variation by sex*
Within the overall pattern, women with thyroid cancer were substantially less likely to be fast-tracked compared with men with the same cancer (odds ratios 0.58, 95% CI 0.50–0.66, *P*<0.0001), whereas women with breast cancer were substantially more likely to be fast-tracked compared with men with breast cancer (odds ratios 1.82, 95% CI 1.60–2.06, *P*<0.0001).
Beyond the two cancers, there was also statistical evidence that women had either higher or lower odds of fast-track referrals than men for another 12 cancers (of the 28 that can occur in either sex), that is, for small intestine, Hodgkin lymphoma, liver and renal cancer (higher) and ALL, stomach, oral, oropharyngeal, mesothelioma, multiple myeloma, laryngeal and rectal cancers (lower).
*Cancer-specific variation by deprivation*
Although the overall pattern of cancer-specific variation by deprivation was small compared with that by age and sex, there was evidence that patients of the most deprived group were more likely than those of the least deprived group to be referred urgently with 4 cancers (small intestinal, oral, vulval and breast), whereas most affluent patients were more likely to be referred under the fast-track route with another 6 cancers (kidney, testicular, bladder, colon, melanoma and lung cancers). There was relatively little variation in patients with the remaining 25 cancers. A similar pattern was seen for cancer-specific variation by deprivation when comparing quintiles 1 and 3 (where the largest difference in main effect size was seen) – there was no evidence for difference between quintiles 1 and 3 for most cancer sites except for endometrial and anal cancer (higher ORs of fast-track referral in quintile 3 compared with the least deprived group).

## Box 2 - Other patterns of variation and likely explanations

*Very old age*. In the oldest patients (above 85 year of age), a slight reduction in the odds of fast-track referrals is observed (*P*<0.0001). This may reflect preferences by patients and clinicians for fewer investigations and ‘routine’ as opposed to fast-track referrals, or the presence of physical or mental health comorbidity (including dementia) that may impede symptom interpretation (NICE, 2015).
*Bladder cancer*. Although previous studies have shown that women with bladder cancer are at increased risk of experiencing diagnostic delay when compared with men (Elliss-Brookes *et al*, 2012; Rogers *et al*, 2014), our findings indicate that doctors are equally likely to use fast-track referral for men and women with that cancer. This seems to suggest that the previously described inequalities by sex in the primary care interval for patients with this cancer (Elliss-Brookes *et al*, 2012; Rogers *et al*, 2014) are likely to occur before a decision to refer is made (i.e., before urological cancer is suspected).
*Thyroid cancer and soft tissue sarcoma*. Another group of cancers with low fast-track referral proportions include thyroid and soft tissue sarcoma that have obvious symptoms in many patients (i.e., abnormal lump) that however have low specificity for cancer. We hypothesise that most patients with thyroid or soft tissue lumps are referred routinely to secondary care unless prior imaging reports exist that suggest otherwise. Ensuring prompt primary care access to ultrasound imaging services may improve initial pick-up rate, whereas the use of one-stop clinics with specialist imaging and biopsy may reduce the overall diagnostic interval for these cancers.
