# Supplementary material for: Variation in ‘fast-track’ referrals for suspected cancer by patient characteristic and cancer diagnosis: evidence from 670 000 patients with cancers of 35 different sites
Source: Br J Cancer. 2017 Nov 28;118(1):24–31. doi: 10.1038/bjc.2017.381 (PMC5765227; doi:10.1038/bjc.2017.381)
Supplement: Supplementary Information [file bjc2017381x1.docx]

**Supplementary File 1. Cancer-specific variation in odds of fast-track referrals by age group (25-34 to 85+).**

**1/3**


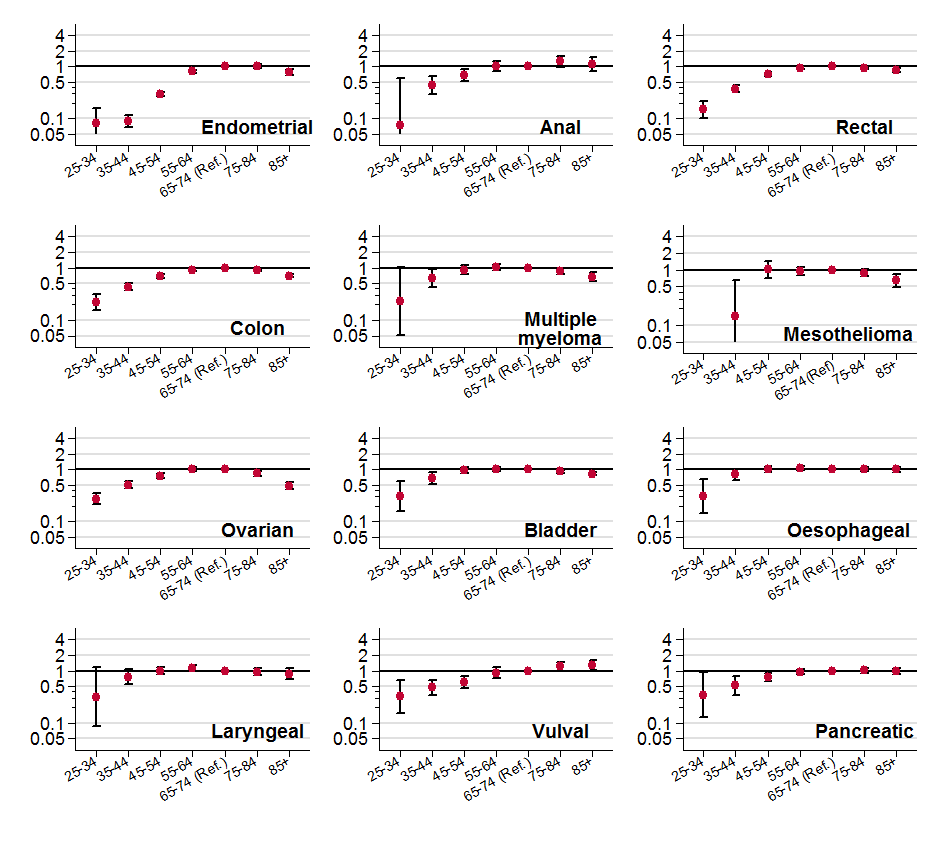


**2/3**


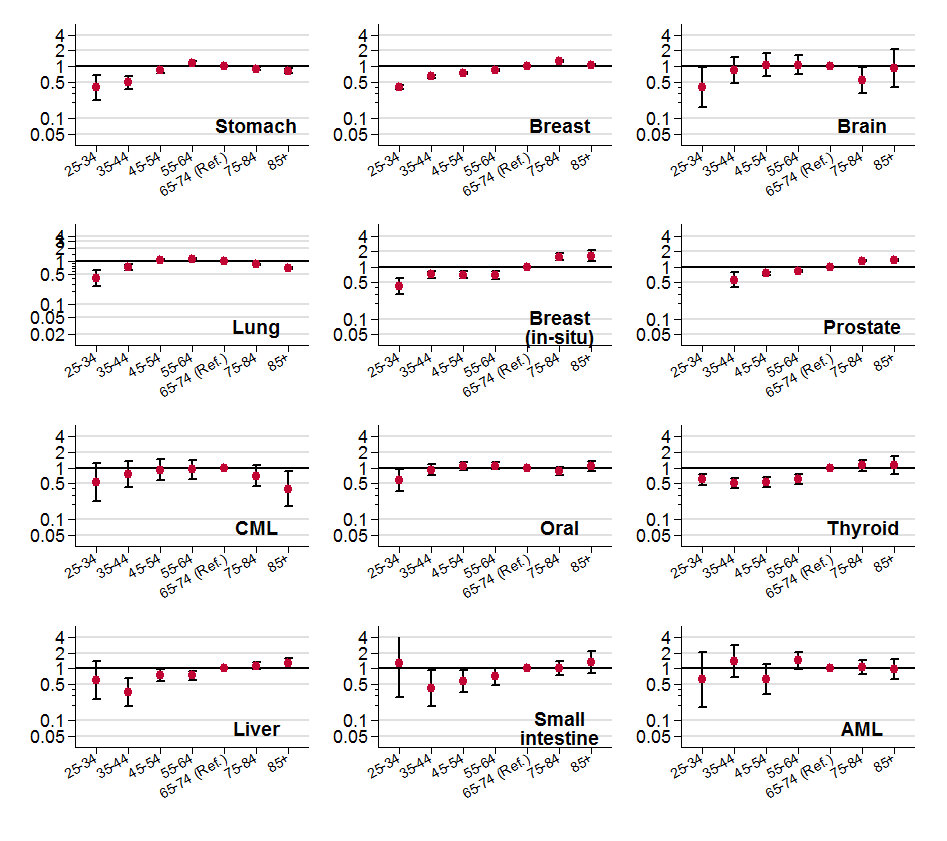


**3/3**


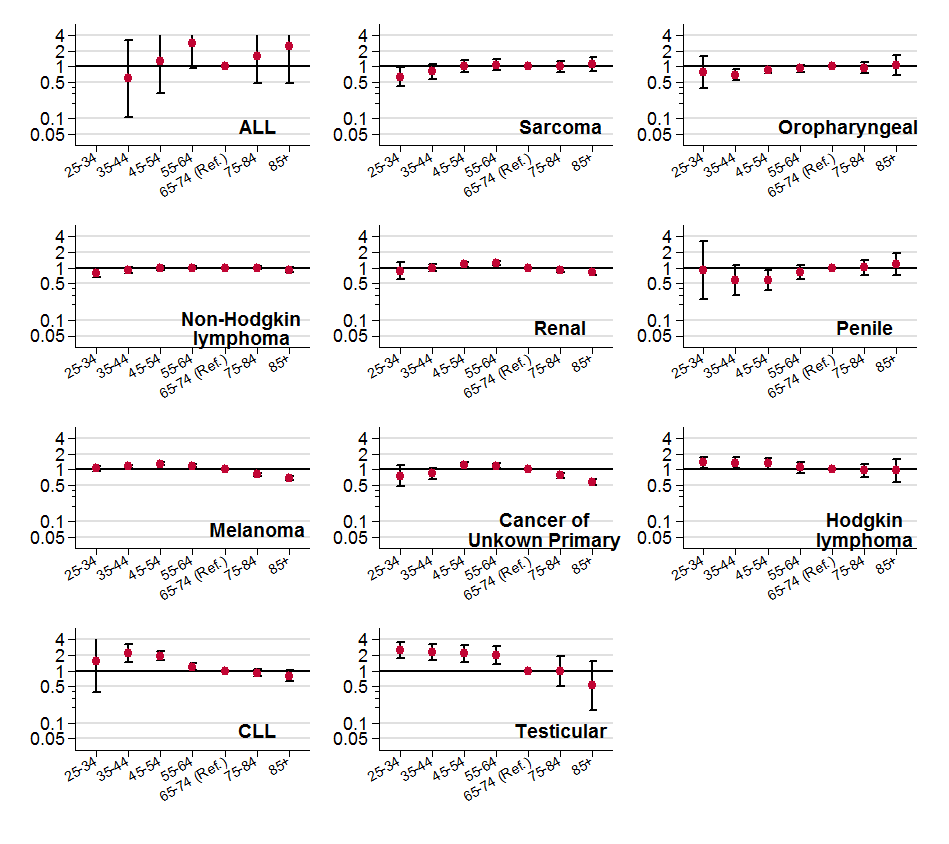


**Supplementary File 2: Age restrictions in NICE 2005 guidelines for individual cancers(2)**

| **Cancer Group as in NICE guidelines** | **Age-related fast-track criteria for referral** | **Age-related fast-track criteria for investigation** | **Age-related non-fast-track criteria for referral** | **Cancers implicated by age-related criteria** | **Correlates with cancer-specific variation by age?** |
| --- | --- | --- | --- | --- | --- |
| Lung | Persistent haemoptysis in smokers >40 |  |  | Lung | No |
| Upper GI |  | Unexplained dyspepsia >55 |  | Oesophageal, stomach | No |
| Lower GI | >40 PR bleed and change in bowel habit |  |  | Colon, rectal | No |
|  | >60 with PR bleed |  |  |  |  |
|  | >60 with change in bowel habit |  |  |  |  |
| Breast | >30F with lump that persists |  | <30F with a lump | Breast | Yes (increasing 2WW with age from 30) |
|  | <30F that enlarges or fixed or family history |  |  |  |  |
|  | >50M with unilateral mass |  |  |  |  |
| Gynaecological | Post-menopausal bleeding |  |  | Endometrial | Yes (large increase in 2WW from 45-54) |
| Urological | 40+ recurrent or persistent UTI with haematuria |  | <50 with microscopic haematuria | Bladder, renal | Possibly (increase in 2WW between 25-34 and 35-44 for bladder), not renal |
|  | 50+ with unexplained microscopic haematuria |  |  |  | No (Not much change from 45-54 onwards) |
| Haematological | No age |  |  |  |  |
| Skin | No age |  |  |  |  |
| Head and Neck | Hoarseness 50+ |  | No age | Laryngeal, Lung | No |
|  | Thyroid swelling 65+ |  |  | Thyroid | Possibly (increase between 55-64 and 65-74) |
| Brain and CNS | No age |  |  |  |  |
| Soft tissue sarcoma | No age |  |  |  |  |
| Children and young people | No age |  |  |  |  |
